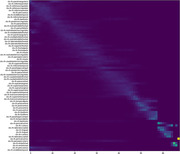# Integrating Connectomics Constraints in Event‐Based Modeling of Alzheimer's Disease Progression: An Analysis of ADNI PET Data

**DOI:** 10.1002/alz.090014

**Published:** 2025-01-09

**Authors:** Mahdieh Vahdatian, Yuji Zhao, Anvar Kurmukov, Boris A Gutman

**Affiliations:** ^1^ Illinois Institute of Technology, Chicago, IL USA

## Abstract

**Background:**

Alzheimer's Disease (AD) presents a major health challenge, with complex and variable neurodegenerative progression. Traditional neuroimaging falls short in fully capturing this heterogeneity. Our study addresses this gap by applying an Event‐Based Model (EBM) to Alzheimer's Disease Neuroimaging Initiative (ADNI) Positron Emission Tomography (PET) data, enriched with connectomics data. This innovative approach promises a more individualized understanding of AD progression, combining PET imaging with insights into brain connectivity from connectomics. Our work aims to enhance early diagnosis and personalized treatment strategies in AD research.

**Method:**

Our study applied an Event‐Based Model (EBM) to Alzheimer's Disease Neuroimaging Initiative (ADNI) datasets across ADNI1, ADNI2, and ADNI3. We analyzed a diverse cohort including Alzheimer's Disease (AD), Mild Cognitive Impairment (MCI), Late MCI (LMCI), Early MCI (EMCI), and cognitively normal (CN) groups, using Amyloid (AMY), Fluorodeoxyglucose (FDG), and Tau PET scans. The methodology integrated connectomics data and employed Monte Carlo Markov Chain (MCMC) techniques for enhanced modeling precision. This approach provided an in‐depth understanding of AD progression, combining advanced statistical analysis with diverse neuroimaging data.

**Result:**

Optimal order of neurodegenerative events ‐ stages of disease progression as measured by changes in metabolic signature (FDG) or Amyloid accumulation ‐ largely followed previously published results. Earliest regions of Amyloid accumulation mirrored the default mode network (Figure 1). Metabolic changes, notably reduction in FDG SUVR, occurred earliest in the same top 10 regions. Also Z‐test statistic (‐4.78): Indicates a significant difference between the average stages of AD and MCI patients, with AD stages being lower than those of MCI.

P‐value (∼0.00000177): Implies an extremely low probability that the observed difference in stages occurred by chance, strongly suggesting a true difference between the groups.

**Conclusion:**

We have presented a novel connectome‐informed progression model of amyloid‐beta accumulation and metabolic changes in the brain. The model discriminates well between stages of cognitive decline and suggests that amyloid accumulation and metabolic changes both follow a similar early of pattern conditioned on white‐matter connectivity